# Long-Term Functional Limitations on Cardiopulmonary Exercise Testing in Emotion-Triggered Takotsubo Syndrome

**DOI:** 10.3390/jcm13041163

**Published:** 2024-02-19

**Authors:** Jean Pierre Jabbour, Luca Arcari, Luca Cacciotti, Damiano Magrì, Tommaso Recchioni, Livia Valeri, Enrico Maggio, Carmine Dario Vizza, Roberto Badagliacca, Silvia Papa

**Affiliations:** 1 Department of Clinical Internal, Anesthesiologic and Cardiovascular Sciences, Sapienza University of Rome, Viale del Policlinico, 155, 00161 Rome, Italy; jeanpierre.jabbour@uniroma1.it (J.P.J.); luca.arcari88@gmail.com (L.A.); tommaso.recchioni@uniroma1.it (T.R.); liviavaleri20@gmail.com (L.V.); enrico.maggio@uniroma1.it (E.M.); dario.vizza@uniroma1.it (C.D.V.); roberto.badagliacca@uniroma1.it (R.B.); 2 Institute of Cardiology, Madre Giuseppina Vannini Hospital, Via di Acqua Bullicante, 4, 00177 Rome, Italy; luca.cacciotti@figliesancamillo.it; 3 Department of Clinical and Molecular Medicine, Sapienza University of Rome, Viale del Policlinico, 155, 00161 Rome, Italy; damiano.magri@uniroma1.it

**Keywords:** cardiopulmonary exercise test, takotsubo syndrome, long-term functional limitations, heart failure

## Abstract

**Background:** In patients with prior Takotsubo syndrome (TTS), long-lasting functional cardiac limitations were described as compared with normal subjects. Emotion-triggered Takotsubo syndrome (E-TTS) has more favorable outcomes than TTS preceded by a physical trigger or by no identifiable factors. The aim of the present study was to assess long-term cardiac functional limitations in a cohort of asymptomatic E-TTS patients. **Methods:** We enrolled *n* = 40 asymptomatic patients with a diagnosis of E-TTS. Cardiopulmonary exercise tests (CPET) were performed at 30 (12–40) months median follow-up from the acute event. A cohort of *n* = 40 individuals matched for age, sex, body mass index and comorbidities served as control. **Results:** Despite recovery of left ventricular ejection fraction, patients with prior E-TTS had lower peak VO_2_ and percentage of predicted peak VO_2_ (17.8 ± 3.6 vs. 22.1 ± 6.5; *p* < 0.001 and 75.2 ± 14.1% vs. 100.6 ± 17.1%, *p* < 0.001), VO_2_ at anaerobic threshold (AT) (11.5 [10.1–12.9] vs. 14.4 [12.5–18.7]; *p* < 0.001), peak O_2_ pulse (9.8 ± 2.5 vs. 12.9 ± 3.5; *p* < 0.001) and higher VE/VCO2 slope (30.5 ± 3.7 vs. 27.3 ± 3.5; *p* < 0.001) compared with matched controls. We found no statistically significant differences in heart rate reserve (HRR), respiratory equivalent ratio (RER), mean blood pressure and peak PetCO_2_ between patients and controls. **Conclusions:** Despite its favorable outcome, patients with E-TTS in our population were found to have subclinical long-term functional cardiac limitations as compared with a control cohort.

## 1. Introduction

Takotsubo syndrome (TTS) is an acute cardiac syndrome, mimicking acute myocardial infarction [1].

TTS is characterized by transient systolic and diastolic left ventricular dysfunction with a variety of wall-motion abnormalities, albeit in the absence of any identifiable culprit such as coronary artery disease (CAD); it mostly affects women and is often associated with a precipitating physical or emotional stressor [1]. Full recovery of left ventricular ejection fraction (LVEF) after days to weeks and myocardial edema in the absence of myocardial scar at cardiac magnetic resonance (CMR) imaging are recognized as key features of TTS [2,3]. However, despite the recovery of LVEF, patients with previous TTS are characterized by a substantial risk of adverse events in the long term [1]. Furthermore, they may display persistent limiting symptoms and can show reduced myocardial strain and increased native T1 mapping values at CMR [4,5] as compared to control subjects. Scally et al. [4] demonstrated that the majority of patients (88%) with previous TTS (>12-month) had cardiac limitations on exercise testing, despite normal LVEF and serum biomarkers. The presence of a physical trigger preceding the event is associated with adverse outcomes both in the short and in the long term [6,7,8]. In these patients, beyond TTS functional abnormalities, the underlying illness contributes to long-term morbidity and mortality, which is mainly non-cardiovascular in these patients [9,10].

Cardiopulmonary exercise testing (CPET) provides a non-invasive assessment of the functional capacity and exercise limitation, including information on individual responses to physical efforts at cardiac and non-cardiac levels [11,12,13]. Indeed, compared with traditional exercise tests, CPET is unique in this way as it can provide a thorough assessment of exercise integrative physiology involving the pulmonary, cardiovascular, muscular, and cellular oxidative systems [14]. Only a single previous study in a population of TTS patients with variable stressful triggers and symptoms burden had attempted to quantify the added value of cardiopulmonary exercise testing (CPET) to a follow-up of TTS patients [4]. Hence, there is uncertainty as to which derived variables and cut-off values to consider.

Based on these premises, we investigated CPET findings in a homogeneous cohort of patients with prior emotion-triggered TTS (E-TTS) and completely recovered LVEF.

## 2. Materials and Methods

We performed an observational case-control study in patients admitted for TTS at Vannini Hospital, Rome, Italy. The Vannini Hospital institution is part of the German–Italian–Spanish Takotsubo (GEIST) registry and shares enrollment criteria and protocols with other participating centers [15]. The diagnosis of TTS was established according to established criteria as previously reported [15]. Briefly, TTS was defined by the presence of (1) transient regional wall motion abnormalities of the left or right ventricle and extending beyond a single epicardial vascular distribution; (2) absence of identifiable culprit coronary artery disease; (3) new and reversible ECG abnormalities; (4) elevated cardiac troponin and natriuretic peptide levels; and (5) recovery of ventricular systolic function at follow-up in all the study patients. All patients underwent coronary angiography to exclude a culprit coronary artery disease (defined as stenosis greater than 50%). Baseline demographics and clinical data were collected, including ECG and echocardiographic presentation. Preceding trigger, if present, was recorded by the physician in charge at the time of hospital admission and reported as emotional, physical or absent as previously described [16]. Clinical, ECG and echocardiographic follow-up protocols in TTS patients at our Institution have been previously described [17].

**Figure 1:**
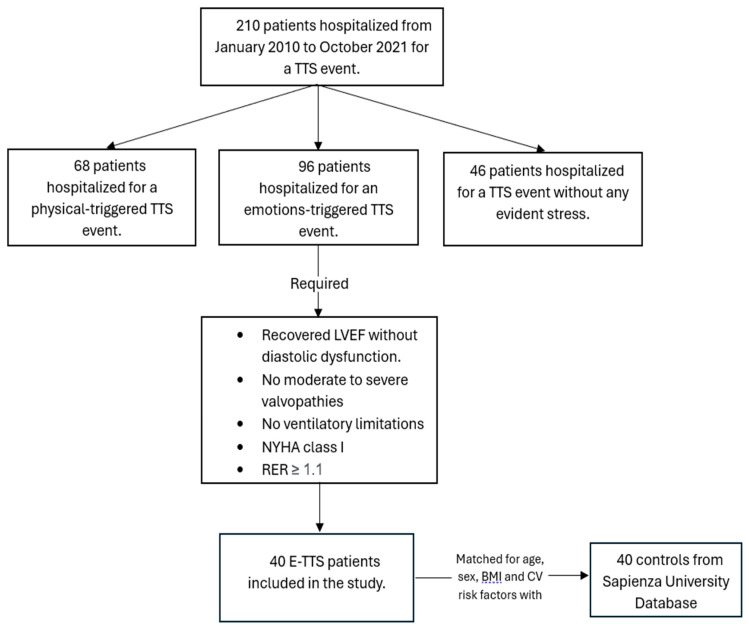
Inclusion of E-TTS patients. BMI = Body Mass Index.

For the purpose of the present study, we retrospectively reviewed our TTS population and included only TTS patients presenting after an emotional trigger (E-TTS) and who had an available CPET. All CPETs in TTS patients were performed at the cardiology unit of the Department of Clinical Internal, Anesthesiological and Cardiovascular Science, Policlinico Umberto I Sapienza University of Rome, as described below. From January 2010 to October 2021, 210 patients had been hospitalized in the Cardiology Department of the Institute M.G. Vannini, for a TTS event. Of them, 96 (46%) patients were hospitalized for an E-TTS, 68 (32%) for a physical-triggered Takotsubo syndrome, and 46 (22%) were diagnosed with a TTS event without any evident stress [18]. Among the E-TTS group, we included in the study those patients with recovered left ventricular ejection fraction and without diastolic dysfunction, moderate to severe valvulopathies nor overt clinical symptoms of dyspnea, who performed an exercise with an RER greater than 1.1 (40 patients). A schematic representation of inclusion of patients is illustrated in Figure 1.

A control group consisted of 40 subjects, recruited from Sapienza University of Rome database and matched for age, sex, body mass index and cardiovascular risk factor distribution and compared with the patients’ cohort. These patients had performed CPET for screening due to a positive familial history of cardiovascular disease. Patients with a forced expiratory volume at end of the first and second measure < 70% of predicted were classified as with ventilatory disorders and were excluded from these analyses.

The study was performed in accordance with the ethical standards as laid down in the 1964 Declaration of Helsinki and its later amendments or comparable ethical standards. Informed consent for the use of clinical data for scientific purposes had been obtained from the patients. The study was approved by the Institutional Review Board for human studies of the Policlinico Umberto I—Sapienza University of Rome, reference number of local ethics committee N° 7502.

### 2.1. Cardiopulmonary Exercise Test

Patients underwent CPET between 1 and 4 years after the acute hospitalization for E-TTS event. All patients receiving beta-blocker drugs were discontinued from their therapy 48 h before performing CPET in order not to affect the VO_2_ peak results [19]. All CPETs were performed in the same daytime condition, without overnight fasting nor a heavy meal just prior to the test, in order to not substantially affect the RER.

All patients performed a symptom-limited incremental CPET. All tests were performed using an electronically braked cycle ergometer (Erg 800S SensorMedics). As standard, before testing we performed CPET flow and gas calibration procedures.

Tests were considered maximal if peak respiratory exchange ratio (RER) was >1.1. Oxygen uptake (VO_2_), carbon dioxide output (VCO_2_), minute ventilation (VE), and end-tidal carbon dioxide partial pressure (PetCO_2_) were measured breath-by-breath (Quark CPET, Rome, Italy) and averaged every 5 s for subsequent analysis. Heart rate (HR) was monitored via 12-lead electrocardiography (ECG). The oxygen pulse was calculated as the VO_2_/HR ratio at peak exercise. Heart Rate Reserve (HRR) was calculated from the difference between predicted peak HR (220—age) and observed peak HR. The anaerobic threshold (AT) was identified by three methods: the V-slope, the ventilatory equivalent, and the end-tidal methods [20]. Peak VO_2_ was defined as the highest VO_2_ that could be sustained for at least 15 s during the last stage of incremental exercise, and the reference values were calculated from the Hansen–Wassermann equations [20]. The slope of VE over VCO_2_ (ΔVE/ΔVCO_2_) during incremental testing was measured from unloaded pedaling to the ventilatory compensation point (VCP). Omitting the latter phase from calculation of the slope made the slope independent of the duration of the test and of the individual’s effort and response to metabolic acid [21]. For patients who did not reach the VCP, it was measured from unloaded pedaling to peak exercise. The dead-space volume of the facemask was subtracted from the total VE before calculating individual VE/VCO_2_ slopes and ratios.

The CPET was safe for all patients, with no adverse events reported during the test.

Before exercise testing, all E-TTS patients underwent basic spirometry in order to calculate the maximum ventilatory ventilation (MVV) to exclude any possible reason of exercise limitation due to ventilatory abnormalities. Informed consent was obtained from each patient before performing CPET.

### 2.2. Statistical Analysis

Normal distribution of variables was assessed by Kolmogorov–Smirnov test. Continuous variables were expressed as mean ± standard deviation. We reported the confidence interval for the mean difference at 95%. Two group comparisons were carried out with t-test for means for independent samples if the data were normally distributed. Non-normally distributed variables were expressed as median and [interquartile range] and compared using Mann–Whitney non-parametric test. After Bonferroni correction, statistical significance was set at *p* < 0.01 in order to reduce the risk of type I errors resulting from multiple hypothesis tests. Categorical variables were expressed as counts and proportions and compared with the Chi-squared test or Fisher’s exact test, as appropriate. All data were analyzed using SPSS Statistics v.26 software (SPSS Inc., Chicago, IL, USA). 

## 3. Results

The median time from acute E-TTS and the CPET was 30 (12–40) months. Patients were predominantly middle-aged (65.2 ± 10.8 years) and women (85%), all with recovered left ventricular ejection fraction. Among E-TTS patients and the control population, the most prevalent comorbidity was hypertension (respectively 37% and 27%; *p* = 0.33), while beta-blockers were the most used medications (respectively 57% and 40%; *p* = 0.11). The most frequently presenting symptom was angina (82%), and the mean LVEF at admission was 41 ± 7%. The most prevalent LV ballooning type was apical (90%), followed by midventricular (10%). Table 1 shows the results of cardiopulmonary exercise testing in patients with E-TTS and in matched control subjects. The demographic characteristics of E-TTS patients and controls at the time of CPET and the characteristics of E-TTS acute events are listed respectively in Table 2 and Table 3.

**Figure 2:**
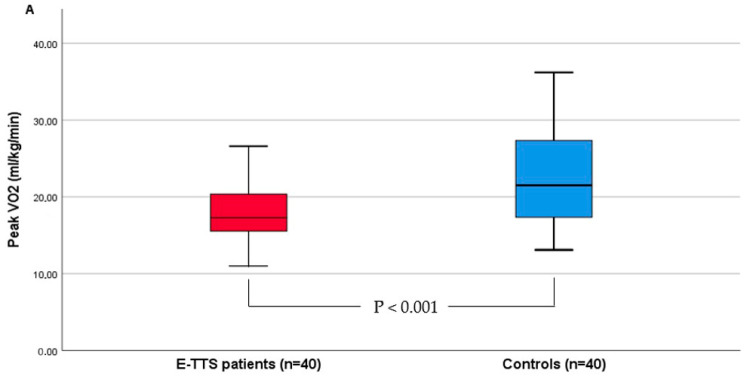

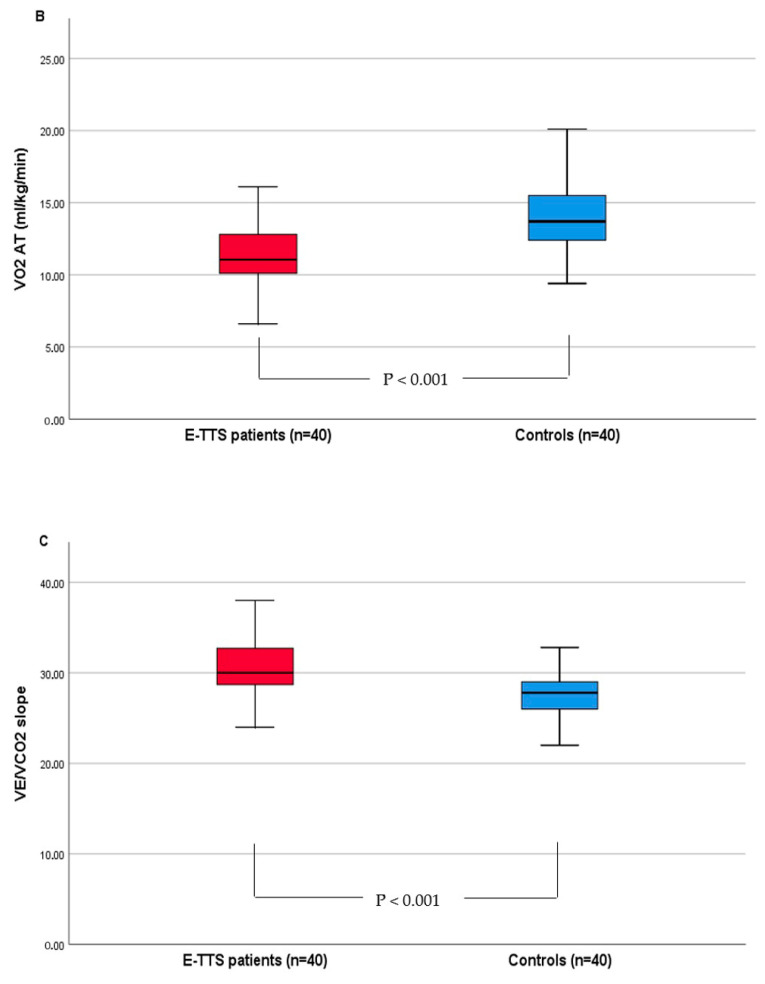

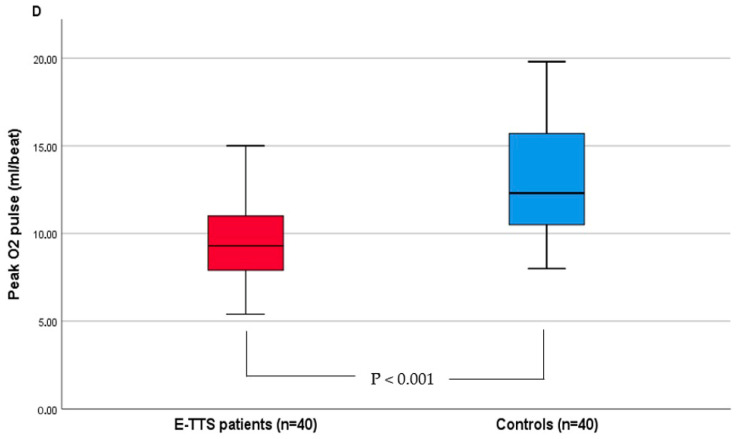
Results of cardiopulmonary exercise test in E-TTS patients and controls. (**A**), peak VO_2_. (**B**) VO_2_ at anaerobic threshold (AT). (**C**), VE/VCO_2_ slope. (**D**), peak O_2_ pulse.

As expected, for CPET, we did not find any sign of exercise reduction due to ventilatory limitation, as shown by breathing reserve, i.e., the relative difference between the maximal voluntary ventilation (MVV) and peak exercise VE (respectively, 47.6 ± 13 vs. 34.1 ± 9.8; *p* < 0.001). Throughout the exercise test, any desaturation event occurred in the two groups. Compared with control subjects, patients with prior E-TTS had lower peak VO_2_ and percentage of predicted peak VO_2_ (17.8 ± 3.6 vs. 22.1 ± 6.5; *p* < 0.001 and 76.2 ± 14.1% vs. 99.9 ± 17.1%, *p* < 0.001), VO_2_ at anaerobic threshold (AT) (11.5 [10.1–12.9] vs. 14.4 [12.5–18.7]; *p* < 0.001), peak O_2_ pulse (9.8 ± 2.5 vs. 12.9 ± 3.5; *p* < 0.001) and higher VE/VCO_2_ slope (30.5 ± 3.7 vs. 27.3 ± 3.5; *p* < 0.001) compared with matched controls (Figure 2). We did not find any statistically significant difference between E-TTS patients and controls in heart rate reserve (HRR) (33.3 ± 19.4 vs. 26.5 ± 13.8; *p* = 0.07), resting systolic blood pressure (120 [110–130] vs. 120 [110–130]; *p* = 0.98), resting diastolic blood pressure (80 [70–80] vs. 80 [70–90]); *p* = 0.21), peak systolic blood pressure (165 [160–180] vs. 170 [160–180]; *p* = 0.46), peak diastolic blood pressure (100 [90–100] vs. 100 [90–100]; *p* = 0.3) peak end-tidal Pco_2_ (36 ± 4 vs. 37 ± 5; *p* = 0.4) and respiratory equivalent ratio (1.12 [1.1–1.19] vs. 1.13 [1.1–1.2]; *p* = 0.6). 

## 4. Discussion

In the present study, we described how asymptomatic NYHA (New York Heart Association functional classification for classifying heart failure in one of four categories based on limitations or symptoms such as dyspnea and angina during physical activity) functional class I patients with a previous episode of E-TTS > 1 year had lower peak VO_2_ and VO_2_ pulse, lower VO_2_ at AT and higher VE/VCO_2_ slope values as compared with matched controls. This highlights the ability of CPET to identify an impaired cardiovascular response to physical activity in the long-term after the acute event, even in the low-risk and asymptomatic E-TTS population with recovered LVEF.

Long-term effects directly attributable to a TTS attack remain controversial due to the paucity of available data and concomitant presence of comorbidities, acute diseases and other confounding factors that can impact the outcomes within this heterogeneous population. Accordingly, multiple studies showed that patients with events triggered by emotional stress had better short and long-term outcomes compared with TTS secondary to physical triggers [7,10,16]. Scally et al. [4] showed a reduction in peak VO_2_ and VO_2_ pulse and an increase in VE/VCO_2_ slope at 20 (13–39) months from the index event in a heterogeneous population of 20 patients with previous TTS compared with matched control subjects. Of note, in that study, approximately one third of the TTS patients did not have an identifiable emotional trigger, a marker of worse prognosis which identifies a subset of high-risk comorbid patients [7]. Furthermore, that mixed cohort of patients was still symptomatic long-term after the acute event, with the majority in NYHA functional class I-II. Our results build on previous evidence and reiterate the concept that functional impairment can be found long-time after a previous TTS episode, even in patients with emotional trigger-only TTS.

In a recent prospective case-control study, Schweiger et al. compared 24 age- and sex-matched TTS-recovered patients with 24 acute coronary syndrome patients, without LVEF residual dysfunction nor respiratory diseases, in terms of performance on CPET from one to six months after the index event; the values of peak VO_2_ and peak Mets were comparable between the two groups (respectively 20.2 ± 5.4 vs. 18.9 ± 7.6, *p* = 0.47 and 5.7 ± 1.6 vs. 5.5 ± 1.9, *p* = 0.68), while there was a trend towards a lower oxygen pulse and higher oxygen cost of work in TTS patients compared to ACS patients (respectively 9.4 ± 2.2 vs. 10.9 ± 3.1, *p* = 0.055 and 12.9 [11.3–15.5] vs. 10.9 [9.7–12.2], *p* = 0.019). It should be noted that all CPETs parameters were considered for subsequent analysis only in those who achieved an RER of at least 1.05 [22]. Moreover, TTS patients were more frequently complaining of persistent symptoms and a subjective low physical fitness compared to ACS patients. Of note, in that study, the nature of the acute trigger of the Takotsubo event was not specified, nor were the comorbidities in TTS and ACS populations.

Our results in terms of lower peak VO_2_ and VO_2_ pulse are comparable to those found by Schweiger et al. When compared with reference values [23], the E-TTS patients in our population showed a slightly lower percentage of predicted peak VO_2_ and absolute peak VO_2_ values, a lower peak O_2_ pulse and VO_2_ AT, and slightly higher VE/VCO_2_ slope values. Lower peak VO_2_ and VO_2_ pulse, VO_2_ at AT, and higher VE/VCO_2_ slope values without ventilatory limitations are all features of a heart failure (HF) phenotype [24], with several studies showing that peak VO_2_ and VE/VCO_2_ slope are cardiovascular predictors of morbidity and mortality in HF of different etiologies [25,26,27,28], such as coronary artery disease, dilated cardiomyopathy, valvular disease and others. The abnormalities found in our E-TTS population were less evident than those in heart failure with reduced ejection fraction (HFrEF) populations, as shown by Agostoni et al. in their population from the MECKI Score multicenter registry, possibly explaining the low symptom burden referred to by our E-TTS patients. Notably, they found a peak VO_2_ of 14.4 ± 4.4 mL/kg/min, (peak VO_2_% of predicted 52.9% ± 15.8%), a VO_2_ AT of 10.1 ± 3.2 mL/kg/min, a peak O_2_ pulse of 9.0 ± 3.1 mL/bpm, and a VE/VCO_2_ slope of 33.0 ± 7.7 in a HFrEF population (mean LVEF of 30.8% ± 9.1) with a mean age of 60.3 ± 12.4 and an NYHA class of 2.2 ± 0.6 [29]. Interestingly, our absolute and percentage of predicted peak VO_2_ and VE/VCO_2_ slope results are comparable to those found by Nadruz et al. in a population of 195 patients diagnosed with heart failure with preserved ejection fraction (HFpEF) with a mean age of 56 ± 15 and with most patients in NYHA class I and II. Notably, they also found that in that population, with a preserved left ventricular ejection fraction, peak VO_2_ and VE/VCO_2_ slope values had incremental prognostic value for the composite of all-cause death, LVAD implantation or heart transplant, and for incident HF hospitalization [27].

Our findings highlight how CPET abnormalities can be detected even in the low-risk and asymptomatic E-TTS subset long after the acute event, and hence suggests that this non-invasive tool could aid in a more comprehensive characterization of TTS patients across the whole clinical spectrum, potentially providing an evidence-based reason to prospectively follow-up this subset of patients or to continue the angiotensin-converting enzyme inhibitor (ACEi)/angiotensin receptor blockers (ARBs)+β-blockers-based long-term treatment for TTS. Notably, a recent review [30] of the current literature has shown conflicting results on the prevention of TTS-event recurrence and amelioration of survival in TTS patients on ACEi/ARB+β-blockers treatment [31]. Our preliminary results support the further investigation of CPET in this clinical context; however, they leave several areas of uncertainty. Firstly, detected abnormalities were not clearly pathologic, and it cannot be excluded that they preceded the development of the TTS event rather than being its consequence, in keeping with the highly vulnerable background of this population related to a clustering of pre-existing factors possibly determining vulnerable phenotypes prone to heart failure [32]. On the other hand, it has been previously hypothesized that TTS itself could have long-term consequences affecting the myocardial interstitium, symptoms and functional capacities of the patients. This has been shown in a TTS population within the most severe spectrum of the disease by using myocardial deformation analysis and T1 mapping cardiac magnetic resonance imaging [4,5]. The relevance of CPET, where mortality in the long-term is mainly driven by non-cardiovascular events [9], remains to be assessed. Indeed, it was observed that most patients with TTS, after the acute phase of the disease, died for causes linked to comorbidities such as cancer [33] chronic kidney disease [34], diabetes mellitus [35] and respiratory disorders [36] that all together can have a relevant impact on prognosis beyond the TTS episode [9]. Notwithstanding, a recent study highlighted that TTS patients might experience an excess of cardiovascular mortality, and that although lower than that of patients with a previous AMI, it remains higher than matched controls [37]. In this context, a thorough cardiac evaluation including CPET could help shed light on the still-unknown role of cardiovascular outcomes in the long-term after a TTS event, which can have an impact on disease specific treatments [38].

### Study Limitations

Our study is partially retrospective and observational, with a relatively limited sample size. Notwithstanding, to the best of our knowledge, this is the largest sample size in the literature comparing TTS patients with a matched control population at a long-term follow-up in terms of CPET performance. Despite peak VO_2_ having been consistently found to be a robust prognostic variable in heart failure patients, it might be underestimated due to reduced patient effort; the inclusion of only CPETs with an RER greater than 1.1 should strengthen the value of a reduced peak VO_2_ by excluding those patients with a lower effort. Of note, VO_2_ at AT and VE/VCO_2_ slope below the VCP are variables in the submaximal range that showed a prognostic value in patients with chronic heart failure [39]. Recollection of the trigger of the acute event by the patients can be a source of recall bias that we cannot exclude in our study. However, we accurately reviewed medical records to exclude all TTS possibly related to medical conditions, procedures, or neurologic disorders [7].

## 5. Future Perspectives

We think our study can pave the way for new prospective longitudinal studies aiming at evaluating the benefit of long-term follow up and ACEi/ARBs + β-blockers therapy maintenance in those asymptomatic E-TTS patients with long-term functional limitations detected during CPET. In the future, we seek to assess the prognostic significance of those CPET functional abnormalities in a larger cohort of E-TTS individuals.

## 6. Conclusions

Despite their overall favorable outcome, asymptomatic NYHA class I patients with E-TTS were found to have long-term subclinical functional cardiac impairments when compared with a matched control population. In particular, they were found to have lower peak VO_2_ and VO_2_ pulse, lower VO_2_ at AT, and higher VE/VCO_2_ slope values at a maximal cardiopulmonary exercise test.

CPET proved to be a useful tool in the long-term evaluation of E-TTS patients after recovery of LVEF, paving the way to prospectively follow-up this subset of patients to promptly detect the appearance of future clinical cardiac impairments. Further studies are needed to fully clarify the origin of the detected CPET abnormalities as well as their prognostic relevance in patients with recovered E-TTS.

## Figures and Tables

**Table 1:** Cardiopulmonary Exercise Testing in Patients With E-TTS and Matched Control Subjects.

	Recovered E-TTS Patients	Matched Control Subjects	*p* Value
Peak VO_2_, mL/min/kg	17.8 ± 3.6	22.1 ± 6.5	<0.001
% predicted peak VO_2_	76.2 ± 14.1	99.9 ± 17.1	<0.001
VO_2_ at AT, mL/min/kg	11.5 [10.1–12.9]	14.4 [12.5–18.7]	<0.001
Peak O_2_ pulse, mL/beat	9.8 ± 2.5	12.9 ± 3.5	<0.001
VE/VCO_2_ slope	30.5 ± 3.7	27.3 ± 3.5	<0.001
Heart Rate Reserve, beats	33.3 ± 19.4	26.5 ± 13.8	0.07
Resting systolic BP, mmHg	120 [110–130]	120 [110–130]	0.98
Resting diastolic BP, mmHg	80 [70–80]	80 [70–90]	0.21
Peak systolic BP, mmHg	165 [160–180]	170 [160–180]	0.46
Peak diastolic BP, mmHg	100 [90–100]	100 [90–100]	0.30
Peak PetCO_2,_ mmHg	36 ± 4	37 ± 5	0.40
RER	1.12 [1.1–1.19]	1.13 [1.1–1.2]	0.60

All data are shown as mean + SD or median and interquartile range as appropriate. VO_2_ = Volume of Oxygen; AT = Anaerobic Threshold; VE = Minute Ventilation; VCO_2_ = Volume of Carbon Dioxide; BP = Blood Pressure; RER = Respiratory exchange ratio.

**Table 2:** Characteristics of E-TTS patients and controls at the time of CPET.

	Patients with Prior E-TTS (40)	Control Subjects (40)	*p*
Age, y (Mean ± SD)	65.2 ± 10.6	64 ± 11.3	0.59
BMI, kg/m^2^	23.6 ± 3.5	23.8 ± 2.6	0.79
Female, *n* (%)	34 (85)	34 (85)	>0.99
Time since acute TTS event (months)	30 (12–40)	-	-
Comorbidities			
Hypertension, *n* (%)	15 (37)	11(27)	0.33
Dyslipidemia, *n* (%)	13 (37)	11 (27)	0.34
Diabetes mellitus, *n* (%)	6 (15)	4 (10)	0.72
Current smoker, *n* (%)	2 (5)	1 (2)	0.55
Psychiatric disease	2 (5)	0	0.32
History of cancer	2 (5)	0	0.32
Medications			
Beta-blocker	23 (57)	16 (40)	0.11
ARBs/ACE inhibitors	22 (55)	13 (32)	0.056
Statin	14 (35)	9 (22)	0.21
Echocardiography			
LVEF, %	60 [55–60]	60 [55–65]	0.66

All data are shown as mean + SD, as median and range a or as number and percentage as appropriate. BMI = Body Mass Index; ARBs = Angiotensin-Receptor Blockers; ACE = Angiotensin-Converting Enzyme; LVEF = Left Ventricular Ejection Fraction.

**Table 3:** Characteristics of acute E-TTS events.

Acute E-TTS Event	
LV ballooning type, *n* (%)	
Apical	36 (90)
Midventricular	4 (10)
ECG on admission	
ST elevation, *n* (%)	26 (65)
ST depression, *n* (%)	6 (15)
Negative T wave, *n* (%)	7 (17)
Presenting symptoms	
Angina	33 (82)
Dyspnea	5 (13)
Echocardiography at presentation	
Left ventricular EF% (Mean ± SD)	41 ± 7

All data are shown as mean ± SD or as number and percentage as appropriate. LV = Left Ventricle; EF = Ejection Fraction.

## Data Availability

The data presented in this study are available on request from the corresponding author.

## Abbreviations

TTS	Tako-tsubo syndrome
AMI	Acute myocardial infarction
LVEF	Left ventricular ejection fraction
CMR	Cardiac magnetic resonance
CPET	Cardiopulmonary exercise testing
E-TTS	Emotions-triggered Tako-tsubo syndrome
GEIST	German-Italian-Spanish Tako-tsubo registry
RER	Respiratory exchange ratio
VO_2_	Oxygen uptake
VCO_2_	Carbon dioxide output
VE	minute ventilation
PETCO_2_	end-tidal carbon dioxide partial spressure
HR	heart rate
VO_2_/HR	oxygen pulse
VCP	Ventilatory compensation point
MVV	maximum voluntary ventilation
AT	anaerobic threshold
HF	heart failure
HFrEF	heart failure with reduced ejection fraction
